# PlaD: A Transcriptomics Database for Plant Defense Responses to Pathogens, Providing New Insights into Plant Immune System

**DOI:** 10.1016/j.gpb.2018.08.002

**Published:** 2018-09-26

**Authors:** Huan Qi, Zhenhong Jiang, Kang Zhang, Shiping Yang, Fei He, Ziding Zhang

**Affiliations:** 1 State Key Laboratory of Agrobiotechnology, College of Biological Sciences, China Agricultural University, Beijing 100193, China; 2 Jiangxi Key Laboratory of Molecular Medicine, The Second Affiliated Hospital of Nanchang University, Nanchang 330006, China; 3 Department of Plant Pathology and the Ministry of Agriculture Key Laboratory for Plant Pathology, China Agricultural University, Beijing 100193, China; 4 Biology Department, Brookhaven National Lab, Upton, NY 11967, USA

**Keywords:** PlaD, Plant, Defense response, Transcriptomics database, Gene expression analysis

## Abstract

High-throughput transcriptomics technologies have been widely used to study **plant** transcriptional reprogramming during the process of plant **defense responses**, and a large quantity of gene expression data have been accumulated in public repositories. However, utilization of these data is often hampered by the lack of standard metadata annotation. In this study, we curated 2444 public pathogenesis-related gene expression samples from the model plant Arabidopsis and three major crops (maize, rice, and wheat). We organized the data into a user-friendly database termed as **PlaD**. Currently, PlaD contains three key features. First, it provides large-scale curated data related to plant defense responses, including gene expression and gene functional annotation data. Second, it provides the visualization of condition-specific expression profiles. Third, it allows users to search co-regulated genes under the infections of various pathogens. Using PlaD, we conducted a large-scale transcriptome analysis to explore the global landscape of gene expression in the curated data. We found that only a small fraction of genes were differentially expressed under multiple conditions, which might be explained by their tendency of having more network connections and shorter network distances in gene networks. Collectively, we hope that PlaD can serve as an important and comprehensive knowledgebase to the community of plant sciences, providing insightful clues to better understand the molecular mechanisms underlying plant immune responses. PlaD is freely available at http://systbio.cau.edu.cn/plad/index.php or http://zzdlab.com/plad/index.php.

## Introduction

Plant diseases caused by pathogens seriously affect food security and might even threaten human health. Fundamental research on the molecular mechanisms of plant immune system plays important roles in continuously improving our knowledge on plant resistance to various pathogens. During the infection of pathogens, plants trigger pattern-triggered immunity (PTI) and effector-triggered immunity (ETI), including a number of immune responses such as hypersensitive response, reduction in reactive oxygen species, as well as activation of mitogen-activated protein kinase (MAPK) cascades or calcium-dependent protein kinases and hormonal modulation [1]. A lot of studies have been devoted to investigating the transcriptional reprogramming related to plant immunity using high-throughput technologies. As a result, public databases such as NCBI Gene Expression Omnibus (GEO) [2] host thousands of expression samples related to plant immune processes.

Transcriptome data bring great opportunities and challenges to explore the molecular mechanisms of plant immunity. So far, many methods have been developed to analyze transcriptome data, such as differential expression analysis [3], [4], [5], gene co-expression analysis [6], and gene differential co-expression analysis [7]. Moreover, the integration of transcriptome data with biological networks often leads to new biological findings [8], [9]. For instance, Dong et al. employed a machine learning method to integrate transcriptional data with gene networks to study PTI and ETI in the context of network biology [10]. Jiang et al. integrated transcriptional data and protein–protein interaction (PPI) network to compare plant defense responses to pathogens with different lifestyles [11]. In general, gene differential expression analysis remains the most popular and direct approach to process transcriptome data related to plant defense responses [12], [13], [14], [15], and the detection of differentially expressed genes (DEGs) has become an effective way to screen plant immunity-related candidate genes.

Existing gene expression databases have played important roles in accelerating the study of gene functions. GEO and ArrayExpress [16] are probably the two main repositories of high-throughput gene expression data. Additional resources or tools have also been further developed to facilitate the expression data analysis. For instance, Expression Atlas [17] provides gene expression analysis across multiple species and biological conditions. GEO2R is a web application of GEO helping users to identify and visualize DEGs. However, to use GEO2R, users have to manually divide samples into several groups and convert probe IDs of some platforms to gene IDs, which is not user-friendly for non-experts. Co-expression analysis of genes has been effective in providing in-depth functional hypotheses of genes [18], [19], [20]. To take full advantage of the currently available expression data, several specialized co-expression databases have also been developed, such as COEXPEDIA [21] and GEM2Net [22]. COEXPEDIA is a co-expression database for humans and mice, with the core idea of inferring co-expression relationships from individual studies, whereas GEM2Net focuses on the co-expression of genes involved in response to biotic and abiotic stresses in Arabidopsis.

Despite the availability of the aforementioned resources, utilization of a large amount of public expression data is not a trivial task. On the one hand, the expression abundance values may not be directly comparable across different studies due to the different experimental designs. On the other hand, public expression data are not annotated using standard ontology, making automatic parsing process not straightforward [23], [24]. In this context, development of specialized transcriptomics databases is still highly required.

Several transcriptomics databases have also been reported for plant immune response. For instance, PathoPlant [25] is a transcriptomics database for analyzing co-regulated genes in plant defense responses. Unfortunately, PathoPlant only contains a small number of expression data of Arabidopsis, thus limiting its application in the plant community. ExPath [26], [27] is also a plant transcriptomics database, which collects more than 1000 samples in biotic stress, abiotic stress, and hormone secretion. It also provides diverse analyses including co-expression analysis, DEG identification, and enrichment analysis of pathways. However, the pathogenesis-related expression data in ExPath is generally not sufficient. It is worth mentioning that although microarray technique might be replaced by direct mRNA sequencing in the foreseeable future, the current pathogenesis-related transcriptome data generated using the microarray technique still hold great value for potential biological discoveries. Therefore, integration of these data for further exploration remains an important task.

In this work, we constructed a user-friendly knowledgebase called PlaD, which contains 2444 curated pathogenesis-related transcriptome samples from 94 GEO series for four plant species, including Arabidopsis, maize, rice, and wheat. Firstly, we quantified the fold change (FC) of gene expression within each study, making data comparable across studies or species. We provided the visualization of the corresponding expression profiles for the condition-specific DEGs. Subsequently, we seamlessly integrated functional annotations of each DEG into PlaD, such as orthologous genes, co-expressed genes, protein interactions, transcriptional regulations, pathways, and protein domains. Finally, a personalized and advanced tool was provided to allow users to search co-regulated genes. To further explore the global gene expression patterns of the curated data, we performed a large-scale transcriptional analysis. We show that only a small fraction of genes are differentially expressed under multiple conditions, revealing their frequent responsiveness to pathogen attacks. Functional analyses of those genes indicate their important roles in plant immunity. Interestingly, it seems that their frequent responsiveness to pathogen attacks might be explained by their tendency of having more network connections and shorter network distances in gene networks.

## Methods

### Data collection and classification

#### Microarray dataset collection and classification

Pathogenesis-related microarray expression datasets of Arabidopsis, maize, rice, and wheat were collected from GEO. In total, we obtained a microarray dataset consisting of 94 series and 2444 samples from four plant species (Arabidopsis, maize, rice, and wheat). These series were then classified based on plant tissues and pathogen types.

#### Gene ID conversion

The gene probes of Arabidopsis, rice, and maize were converted into TAIR [28], RAP-DB [29], and MaizeGDB [30] gene locus IDs, respectively. If multiple probe sets were mapped to the same gene, the corresponding gene ID was assigned to the probe with the largest expression variance. Finally, the gene IDs of 33,309 Arabidopsis genes, 29,695 rice genes, and 13,280 maize genes were mapped. For wheat, we downloaded cDNA sequences from Ensembl Plants (http://plants.ensembl.org/Triticum_aestivum/Info/Index; release 32) and used Blastn [31] to map probes to genes [32]. According to the strict thresholds with *E*-value <1E−05, sequence identity >0.97, and global coverage >0.98, 7782 probes were mapped to 6535 wheat genes.

### Differential expression analysis

To identify condition-specific DEGs, we grouped expression samples within series according to plant ecotypes, genotypes, and infection status by pathogens. DEGs were calculated for each comparative condition within the same series. After normalization and log_2_ transformation of expression values, DEGs were inferred through the R package “limma” [33] with absolute log_2_FC ≥1.5 and false discovery rate (FDR)-adjusted *P* value <0.05.

### Co-expression network construction

The construction of condition-specific co-expression networks was based on individual series rather than the combined expression data from multiple series. If more than one pathogen were involved in one series, co-expression networks were inferred for each pathogen. Only series containing at least 12 samples were taken into account. For each series, we calculated the adjacency value between any two genes using the signed WGCNA co-expression measure [34]. Then, the top 0.1% pairs ranked according to the adjacency values were selected as co-expressed gene pairs. For each gene with ≥5 co-expressed genes, gene ontology (GO) enrichment analysis was further performed for the co-expressed genes.

### Gene information collection and functional annotation

Genes detected by microarray analysis were annotated and displayed on our website. Gene information collection procedures and functional annotation methods of Arabidopsis, rice, wheat, and maize are detailed as follows:

#### Arabidopsis and rice genes

(i) Gene short descriptions, GO annotations, as well as protein sequences of Arabidopsis and rice were downloaded from TAIR and RAP-DB, respectively. (ii) Transcriptional regulatory interactions of Arabidopsis genes were retrieved from ATRM [35], AGRIS [36], and AthaMap [37]. (iii) Metabolic pathways were obtained from AraCyc [38] and OryzaCyc in Plant Metabolic Network [39]. The annotation of Kyoto Encyclopedia of Genes and Genomes (KEGG) pathways [40] was also collected. (iv) The PPI data of Arabidopsis was collected from BioGRID [41], IntAct [42], and TAIR. (v) The domains of protein sequences were assigned using InterProscan searching [43] against Pfam [44] and SMART [45]. (vi) The corresponding co-expression subnetwork for each gene was constructed using the signed WGCNA. (vii) The conditions under which the gene was differentially expressed were summarized for each gene. The associated conditions were categorized into different classes according to pathogen types.

#### Wheat genes

The protein sequences were downloaded from Ensembl Plants. The gene short descriptions and GO annotations were downloaded from Ensembl Plant Biomart [46]. The annotations of protein domain and differential expression condition were conducted using the same methods as for Arabidopsis.

#### Maize genes

(i) Protein sequences were downloaded from MaizeGDB. (ii) Gene brief descriptions, GO annotations, and metabolic pathways were downloaded from Gramene [47]. The annotations of protein domains, co-expression networks, and differential expression condition were conducted using the same methods as for Arabidopsis.

The stand-alone InParanoid program [48] was used to find orthologous genes between any two species of the four plants examined, and the orthologous gene information was also provided in PlaD.

### Definition of consistency_score

When calculating the frequency of differential expression under multiple conditions, we only focused on the impacts of pathogens on plants, that is, the expression change of one gene caused by plant ecotype or genotype is not considered. Genes differentially expressed under at least 10 conditions are defined as frequently DEGs (freq_DEGs). Limited by the number of conditions, we only identified the freq_DEGs of Arabidopsis and rice. Consistency_score was defined as a benchmark for each freq_DEG:

$$\text{consistency}\_\text{score}\;\;\text{=}\;\;\frac{{\text{Num}}_{\text{up}}\;-{\text{Num}}_{\text{down}}}{{\text{Num}}_{\text{up}}{\;\;\text{+}\;\;\text{Num}}_{\text{down}}}$$

where Num_up_ is the number of conditions leading to up-regulated expression of a DEG, and Num_down_ is the number of conditions for the down-regulated expression of a DEG. Based on the calculated consistency_scores, freq_DEGs can be further divided into different groups. The gene is defined as consistently up with consistency_score ≥0.7, whereas a gene is defined as consistently down with consistency_score ≤−0.7. In this study, we mainly focus on the consistently up and consistently down types of freq_DEGs.

### Database construction

The website construction of PlaD was based on CentOS 6.2, Apache 2.2.15, MySQL 5.6.21, and PHP 5.5.19. D3.js is a JavaScript library for manipulating documents and visualizing data. Highcharts.js is a JavaScript library, providing a convenient way to add interactive charts. The visualization of heatmaps and pie charts is implemented in D3.js, and line charts are implemented in highcharts.js. Cytoscape.js [49] is a graph library for visualization, which is used to display co-expression networks in this work.

### Pathway enrichment analysis

The pathway data were downloaded from KEGG. Using all mapped genes as a reference set, over-represented pathways were identified by hypergeometric test, with *P* values adjusted using the Benjamini–Hochberg correction [50].

### Statistical analysis for transcription factors regulating freq_DEGs

Experimental regulatory interactions were collected from AGRIS [36], ATRM [35], and PlantRegMap [51]. After filtering redundant interactions, we obtained 32,711 regulatory interactions.

According to the core idea of the *in silico* regulatory interaction, the regulatory relationship between the known transcription factor (TF) and the gene is established if a gene contains one known regulatory motif. 619 known regulatory motifs corresponding to 619 Arabidopsis TFs were downloaded from PlantTFDB [51]. Each motif was scanned in the region 1 kb upstream of any Arabidopsis gene using FIMO [52] with default parameters. Finally, we obtained 4,702,150 motif-based regulatory interactions and 615 TFs with >5 targets in Arabidopsis.

For the experimentally identified or predicted regulatory interactions between TFs and Arabidopsis genes, hypergeometric test was employed to judge if a TF significantly regulate up- or down-regulated freq_DEGs, with *P* values adjusted using the Benjamini–Hochberg correction.

### Calculation of topological parameters in networks

The node degree of each gene and average distance between two genes in networks were measured using the “igraph” package in R.

## Results and discussion

### A manually curated atlas of gene expression for plant–pathogen interaction

**Figure 1:**
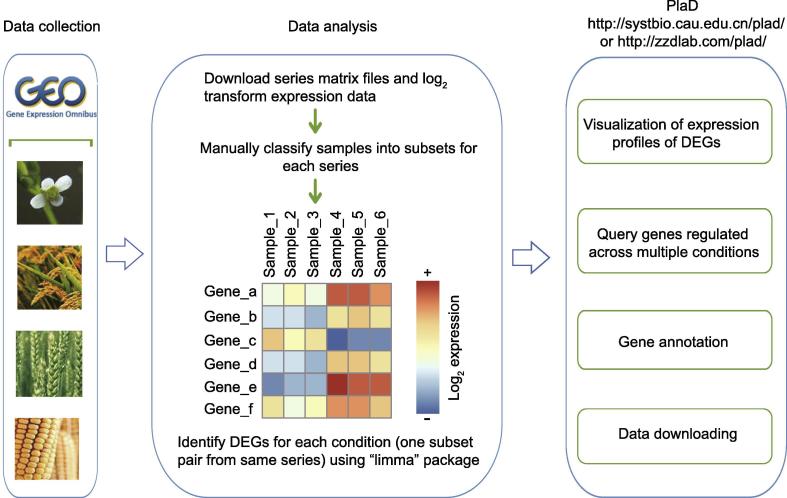
**The flow diagram for constructing the PlaD database** Pathogenesis-related microarray data of Arabidopsis, maize, rice, and wheat were collected from GEO, and series matrix files with pre-processed format were downloaded. To identify DEGs, samples of each series were classified into several subsets based on infection stages, genotypes, and tissues of plants. The “limma” package was used to detect DEGs for subset pairs on a log_2_ scale of gene expression. Note that a subset pair, also defined as a condition, should be from the same series. GEO, Gene Expression Omnibus; DEG, differentially expressed gene.

**Figure 2:**
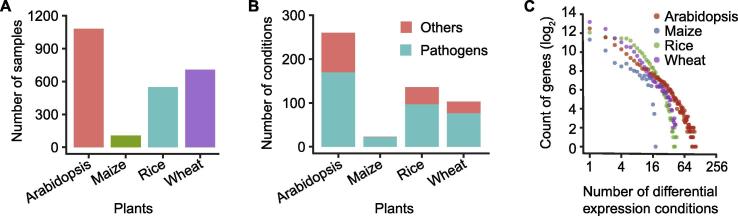
**Data statistics of PlaD** **A.** The number of expression samples for each species covered in PlaD. **B.** The number of differential expression conditions for each species covered in PlaD. Pathogens here represents the “pathogen versus normal” condition, whereas others refer to “pathogen versus pathogen” and “genotype versus genotype” conditions. **C.** The distribution of differential expression conditions for each species covered in PlaD.

The flow chart of our work is shown in Figure 1. We collected 2444 public expression samples from 94 plant pathology-related GEO series, covering four plant species (*i.e.*, Arabidopsis, maize, rice, and wheat) (Tables S1 and S2). All the statistics of PlaD are provided in Figure 2. The corresponding sample size of these four species is 1081, 549, 707, and 107, respectively (Figure 2A). Based on pathogen types and the infected plant tissues, these samples were manually classified into five and thirteen categories, respectively (Figure S1). Each subset represents a group of samples collected from the same tissue and infected by the same pathogen, and two subsets within the same series constitute a comparative condition, such as “pathogen versus normal”, “one pathogen versus another pathogen” and “one genotype versus another genotype”. Then, DEGs were calculated for 522 conditions, 366 of which were “pathogen versus normal” (Figure 2B). DEGs identified from “pathogen versus normal” conditions were considered as pathogen-responsive genes for further analyses. Finally, all of the data were organized into a user-friendly knowledgebase called PlaD.

### Main interfaces and usages of the database

Currently, PlaD is mainly composed of three components, and the corresponding web interfaces and usages are elaborated as follows.

**Figure 3:**
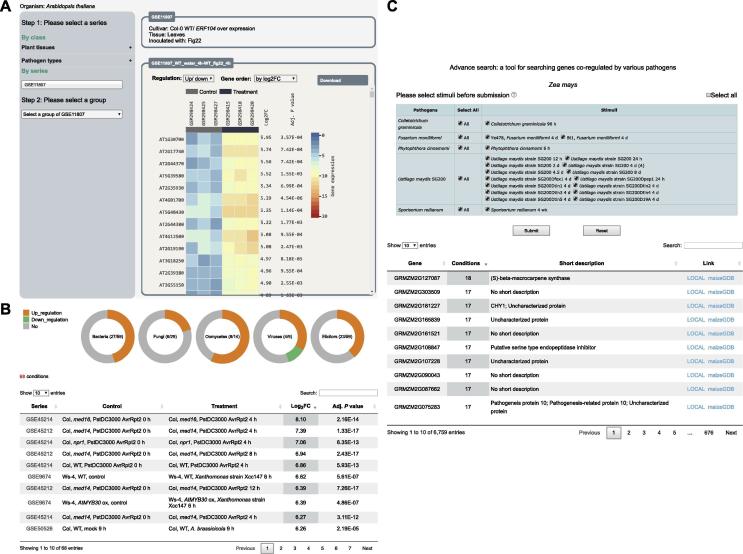
**Three main interfaces of PlaD** **A.** Condition-specific gene expression profiles. **B.** The visualization module of the differentially expressed conditions. **C.** Advanced search for genes co-regulated by various pathogens.

The first and foremost component of PlaD is the presentation of expression profiles (Figure 3A), in which condition-specific DEGs and the corresponding changes in expression level are provided. First, users can select a GSE series via a user-friendly web interface. Here two selection options are offered: by classification and by search. If users select series by classification, two options (pathogen types or infected plant tissues) are further implemented. Once the series is selected, the detailed information of the series will be shown on the upper right of the web page. Second, users should select one condition of their interest. After the selection, the expression profile visualization of DEGs will be shown on the lower right of the page. We offer various ways to show the expression profile, such as selective display of DEGs and different gene order, and also provide the download function of DEGs. The expression profile mainly consists of sample names, sample classification, DEG names, expression value, log_2_FC, and adjusted *P* value. While sample names have external links to GEO, the DEG names are linked to the corresponding gene information pages as described in the following paragraph.

The second component of PlaD is to provide detailed functional information of DEGs. To provide in-depth functional annotation, heterogeneous plant functional data, such as co-expression networks, protein interactomes, transcription regulations, and metabolic pathways are incorporated into this component. Here we only focus on describing the visualization of DEGs and the corresponding co-expression networks. Since the up-regulated and down-regulated genes may be involved in different biological processes or play different roles in plant immunity, we add a visualization module to show the differentially expressed conditions of a gene. The module includes two panels: one demonstrates the number of differentially expressed conditions in different pathogen types, and the other one provides detailed information, such as related series, treatment and log_2_FC, as well as adjusted *P* value (Figure 3B). For each DEG, its associated co-expression network is also provided, and a color scheme based on the consistency_scores of genes is used in the network representation. More importantly, GO enrichment analysis was conducted for co-expressed genes of the query DEG. The enriched GO terms are shown on the right side of the web page. Based on the strategy of “guilt-by-association” [53], the co-expression network may provide some important hints to the potential functions of the query DEG at a systems level.

The third component of PlaD is a personalized and advanced tool that allows users to search genes co-regulated by related stimuli (Figure 3C). After users submit their query, genes that are differentially expressed in at least one condition will be shown. The resultant terms contain related genes, the number of differentially expressed conditions, short descriptions, and links. By default, the result is sorted according to the number of differentially expressed conditions, although it can also be sorted according to gene name or short description. Users can further search the result through keywords. In addition, users can click the provided local or external links for further exploration.

It is of note that currently only the microarray expression data are collected in PlaD. With the accumulation of RNA-seq data, PlaD will be updated to incorporate plant immunity-related RNA-seq data in the future. Thus, PlaD will definitely include more plant species in the future. Since PlaD relies on publicly available data, it inevitably has limitations such as the imbalance of the integrated data in different plant species. For instance, PlaD only provides the PPI information of Arabidopsis due to the insufficiency of experimentally determined PPIs in the other three species included.

### A case study of PlaD application

To illustrate the application of PlaD, we use the Arabidopsis gene AT1G56060 as an example. AT1G56060 encodes a cysteine-rich/transmembrane protein, which is differentially expressed under 68 conditions and shows consistently up-regulated expression after infection. The expression data suggest that AT1G56060 might play an important role in plant immune system. However, the current functional annotation of AT1G56060 is deficient. PlaD also provides its co-expression sub-networks and enriched GO terms based on its local network. We take the co-expression sub-network of AT1G56060 under the infection of *Golovinomyces cichoracearum* as an example. As shown in Figure S2, AT1G56060 is co-expressed with 14 genes, 11 of which are consistently up-regulated. There are 42 enriched GO terms in the “Biological Process” category. Some enriched GO terms are related to plant defense responses, such as “respiratory burst involved in defense response”, “defense response signaling pathway, resistance gene-independent”, “response to salicylic acid”, and ‘response to fungus”. By performing GO enrichment analysis of the co-expressed genes, potential functions of the query gene can be inferred, which provides important functional hypotheses for further experimental validation. Very recently, the functional role of AT1G56060 in response to abiotic stress has been reported [54], and we expect its role in response to pathogen infection to be deciphered in the future. In summary, PlaD can be used to discover candidate genes for plant disease resistance, and to predict potential functions of genes.

### Pathogens trigger large-scale expression changes in plant genes

To demonstrate the biological significance of PlaD, we conducted large-scale transcriptome analyses of the curated data collected in PlaD. We are firstly interested in knowing how many genes may be differentially expressed in response to pathogen attacks (focused on the Arabidopsis data in most of the following analyses, if not specified). We found that approximately 58% (19,366 out of 33,309) of all Arabidopsis genes on the microarray platform was differentially expressed under at least one condition, indicating the complexity of the plant immune gene networks. Although only a handful of core genes are involved in the plant immune process [55], [56], the gene networks might be complicated enough considering that all genes expressed in the relevant cells can affect each other [57].

The results further show that only a small fraction of genes are differentially expressed under multiple conditions. Nonetheless, the fraction was much higher than randomly expected (Figure S3). For instance, 327 genes were simultaneously detected as DEGs under more than 50 conditions (approximately 30% of “pathogen versus normal” conditions in Arabidopsis). The number of genes was significantly higher than that produced by 1000 simulation experiments with randomly selected DEGs (empirical *P* < 0.001). Interestingly, we noticed an approximate power-law distribution of the condition numbers for DEGs (*R*^2^ = 0.91, Figure 2C), possibly reflecting an underlying core network of plant immune process [58]. According to the definition of freq_DEGs, we identified 4762 freq_DEGs in Arabidopsis, which may play important roles in plant–pathogen interaction.

### Freq_DEGs are enriched in plant–pathogen interaction pathways

**Figure 4:**
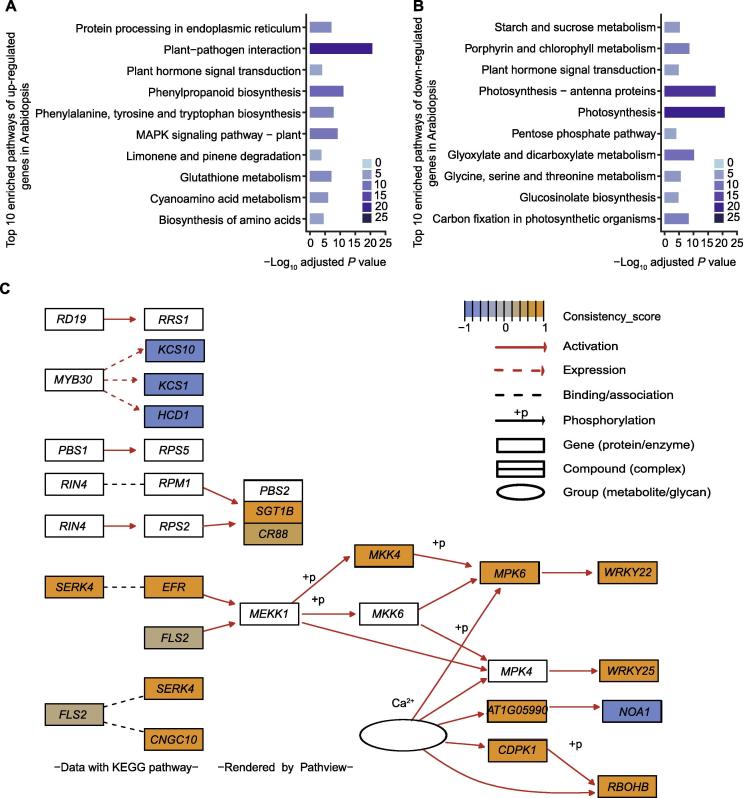
**Enriched KEGG pathways for Arabidopsis** **A.** Top 10 enriched KEGG pathways of 2062 consistently up-regulated freq_DEGs in Arabidopsis under pathogen attacks (consistency_score ≥ 0.7). **B.** Top 10 enriched KEGG pathways of the 1734 consistently down-regulated freq_DEGs in Arabidopsis under pathogen attacks (consistency_score ≤−0.7). **C.** Visualization of the pathway of plant–pathogen interaction. Consistency_scores of freq_DEGs were mapped to the corresponding pathway genes using Pathview. The median value of a node (gene/protein/enzyme or compound) is used if multiple genes were mapped to the same node.

Among the 4672 freq_DEGs in Arabidopsis, expression of 2062 freq_DEGs was consistently up-regulated (consistency_score ≥0.7) under pathogen attacks, whereas expression of 1734 freq_DEGs was consistently down-regulated (consistency_score ≤−0.7). To investigate the functions of freq_DEGs, we performed the KEGG pathway enrichment analysis for the consistently up-regulated and down-regulated genes, respectively. The top 10 enriched pathways are shown in Figure 4A (up-regulated genes) and B (down-regulated genes), and the full list of the enriched pathways is provided in Table S3 (up-regulated genes) and S4 (down-regulated genes), respectively. “Plant–pathogen interaction” is the most enriched pathway for the up-regulated freq_DEGs (ath04626, adjusted *P* = 2.03E−21), containing 52 up-regulated genes (Figure 4C). The R package Pathview [59] was used to map gene consistency_scores to KEGG pathways. EF-TU receptor (EFR) is involved in PTI and is detected as a consistently up-regulated gene in this pathway. As an important kinase in plant–pathogen interaction, the activation of EFR could trigger another up-regulated pathway (*i.e.*, ath04016: MAPK signaling pathway, adjusted *P* = 5.99E−10) to further induce the expression of defense-related genes, such as the genes encoding WRKY29 (consistency_score = 0.93) and PR1 (consistency_score = 0.85). “Phenylpropanoid biosynthesis” is another significantly up-regulated pathway (ath00940, adjusted *P* = 7.36E−12), which is an important pathway in plant immune system. It has been known that phenylpropanoids are precursors to lignin, flavonoids, and stilbenes, and participate in the formation of secondary resistance metabolites [60]. These compounds play important roles in plant defense responses. Pathway “phenylalanine, tyrosine and tryptophan biosynthesis” was also detected as an up-regulated pathway in this study (ath00400, adjusted *P* = 1.48E−08), which is in line with the importance of amino acid metabolism in plant immune responses. These results clearly indicate the important roles of the up-regulated freq_DEGs in the regulation of plant immunity, including the perception of pathogens, activation of the MAPK pathway, and biosynthesis of defense-related products. Meanwhile, we found that down-regulated freq_DEGs were related to metabolisms that affect the growth and development of plants. The most significantly down-regulated pathway is “photosynthesis” (ath00195, adjusted *P* = 1.92E−21), which is consistent with our previous study [61].

Similar result was obtained in rice. For instance, it was found that up-regulated freq_DEGs were enriched in “Phenylpropanoid biosynthesis” (dosa00940, adjusted *P* = 3.33E−07), “Phenylalanine, tyrosine and tryptophan biosynthesis” (dosa00400, adjusted *P* = 4.23E−04), “MAPK signaling pathway” (dosa04016, adjusted *P* = 7.12E−03), and “Plant–pathogen interaction” (dosa04626, adjusted *P* = 4.62E−02), whereas down-regulated freq_DEGs were enriched in “Photosynthesis” (dosa0195, adjusted *P* = 7.74E−06) (Figure S4, Tables S5 and S6).

### Freq_DEGs might be regulated by major TFs

We used the known experimental regulatory data to assign TFs regulating freq_DEGs. We found that 53 TFs significantly regulated the expression of the up-regulated freq_DEGs (hypergeometric test, *P* < 0.01), whereas 33 TFs significantly regulated freq_DEGs with down-regulated expression pattern (hypergeometric test, *P* < 0.01). We compared these 53 TFs with known defense-related TFs collected by Tsuda and Somssich [62], and found that those TFs were significantly enriched in defense-related TFs (hypergeometric test, *P* = 1.14E−03).

Considering that experimentally validated regulatory relationships between TFs and their targets are limited, especially in plants, we conducted *in silico* regulatory motif prediction among the promoter regions of the freq_DEGs. Then, we calculated enriched motifs for the up-regulated and down-regulated freq_DEGs of Arabidopsis. Consequently, we found enriched motifs that correspond to 265 and 132 TFs for the up-regulated and down-regulated freq_DEG, respectively (Tables S7 and S8). The overlap of TFs identified by the experimental approach (Tables S9 and S10) and *in silico* analysis is also significantly high (hypergeometric test, *P* = 3.34E−03 for TFs identified from up-regulated genes, and *P* = 2.57E−03 for TFs identified from down-regulated genes), suggesting the reliability of the *in silico* TF identification. Comparatively, the *in silico* method identified more regulatory relationships between TFs and freq_DEGs, which may provide important clues to facilitate the construction of the regulatory network related to plant immunity. Not surprisingly, defense-related TFs were enriched in 265 TFs that regulate the expression of up-regulated genes (*P* = 2.99E−04). The WRKY TF family consists of 72 proteins in Arabidopsis [51], and previous studies have already proposed that WRKY TFs regulate gene expression in plant defense responses [55]. In this study, all of the 43 WRKY TFs which have more than five targets were identified to regulate the expression of the up-regulated freq_DEGs, demonstrating the important role of WRKY TFs in plant immunity. Similarly, we identified 22 TFs that regulated the expression of up-regulated genes in rice through *in silico* prediction, 21 of which belong to the WRKY TF family.

### Freq_DEGs tend to have specific network properties in gene networks

**Figure 5:**
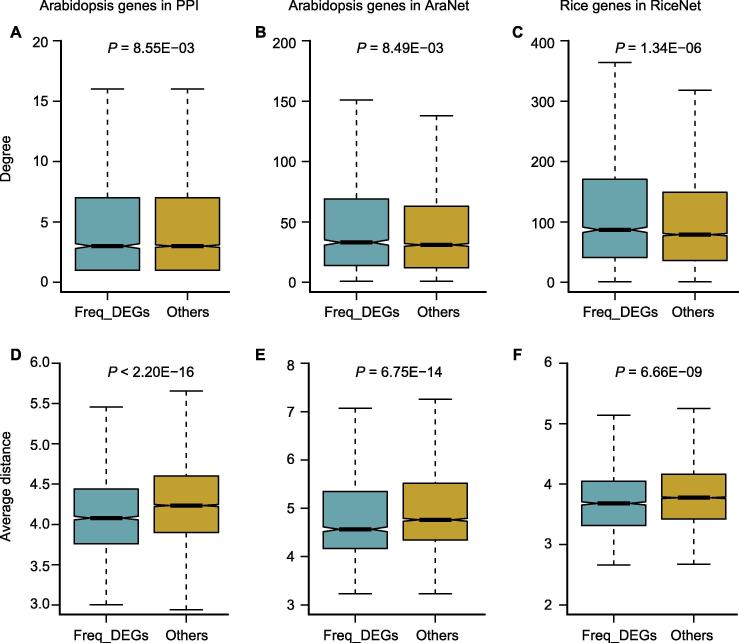
**Network topological analysis of freq_DEGs** Boxplots show the degree difference of freq_DEGs and other genes in the PPI network (**A**), AraNet (**B**), and RiceNet (**C**), respectively. The average distance between freq_DEGs is compared with the average distance between other genes in the PPI network (**D**), AraNet (**E**), and RiceNet (**F**), respectively. The black line in the box indicates the median. The upper and lower edges of the box are the first and third quartiles, respectively.

To further explain why freq_DEGs tend to be frequently differentially expressed under different pathogen conditions, we analyzed their network topologies in the context of gene networks, including one PPI network and two gene co-functional networks. The Arabidopsis PPI data were downloaded from TAIR [28], IntAct [42], and BioGRID [41]. As a result, we obtained a PPI network covering 38,506 non-redundant PPIs. We found that freq_DEGs tended to have higher network degree compared with other genes in the PPI network (Wilcoxon’s rank sum test, *P* = 8.55E−03), indicating that proteins encoded by the freq_DEGs would have a higher chance to be hub proteins and thus play crucial functional roles in the PPI network (Figure 5A). We further examined the network degree of freq_DEGs in two gene co-functional networks using the ‘gold standard’ data downloaded from AraNet v2 [63] and RiceNet v2 [64], which contain co-functional gene pairs for Arabidopsis and rice, respectively. By comparing network degrees between freq_DEGs and other genes in the whole network, we observed higher degrees for freq_DEGs both in Arabidopsis (*P* = 8.49E−03; Figure 5B) and rice (*P* = 1.34E−06; Figure 5C).

To further investigate the network properties of freq_DEGs, we examined the network distances between freq_DEGs. We found that the average distance between freq_DEGs were significantly shorter than other genes in the Arabidopsis PPI network (Wilcoxon’s rank sum test, *P* < 2.20E−16, Figure 5D). Similarly, freq_DEGs were also significantly closer to each other than other genes in two co-functional networks (*P* = 6.75E−14; Figure 5E) for Arabidopsis and rice (*P* = 6.66E−09, Figure 5F), respectively. Such network property could allow freq_DEGs to quickly communicate with each other and thus to achieve effective responses against pathogen attacks, which is in line with our previous analysis on plant immune networks [10], [65]. Collectively, the network topology analyses suggest that freq_DEGs are likely to be involved in more network interactions and to have shorter network distance, partially explaining their frequently differential expression under pathogen attacks.

## Conclusions

Deciphering plant immune response mechanisms is an important research topic in plant sciences. Although a large amount of pathogenesis-related transcriptome data have been released in the past decades, it is still difficult to access these data from public repositories quickly and accurately. In this context, we took initiative to construct PlaD, a comprehensive transcriptomics database. In the meantime, we also conducted exploratory analysis based on the curated transcriptome data in PlaD. Compared with existing similar databases such as PathoPlant and ExPath, PlaD collected and curated more plant pathology-related transcriptomics data. Moreover, we would like to emphasize two key features of PlaD to support customized data mining and in-depth functional annotation. First, it allows users to search co-regulated genes and the corresponding gene activities under the infections of various pathogens. Second, diverse plant functional data, such as co-expression networks, protein interactomes, transcriptional regulations, and metabolic pathways, are also seamlessly integrated into PlaD. Taken together, we hope that PlaD can serve as a user-friendly database to facilitate plant immunity research.

## Authors’ contributions

HQ conducted the study and drafted the manuscript. ZZ and FH supervised the study and revised the manuscript. ZJ, KZ, and SY were involved in data analysis. All authors read and approved the final manuscript.

## Competing interests

The authors have declared no competing interests.

## Appendix A Supplementary material

Supplementary Figure S1 **Classifications of the microarray data A.** The data were classified by pathogen types. **B.** The data were classified by plant tissues.

Supplementary Figure S2 **The co-expression sub-network of the gene AT1G56060 under the infection of *Golovinomyces cichoracearum*** Genes co-expressed with AT1G56060 are shown in the left panel. The right panel shows enriched GO terms among the co-expressed genes.

Supplementary Figure S3 **The number of DEGs differentially expressed under multiple conditions is much larger than randomly expected A.** Arabidopsis. **B.** Rice.

Supplementary Figure S4 **Enriched KEGG pathways for rice A.** Top 10 enriched KEGG pathways of 1496 consistently up-regulated freq_DEGs in rice under pathogen attacks (consistency_score ≥0.7). **B.** Top 10 enriched KEGG pathways of the 1144 consistently down-regulated freq_DEGs in rice under pathogen attacks (consistency_score ≤−0.7).
