# Supplementary figures and images for: PlaD: A Transcriptomics Database for Plant Defense Responses to Pathogens, Providing New Insights into Plant Immune System
Source: Genomics Proteomics Bioinformatics. 2018 Sep 26;16(4):283–93. doi: 10.1016/j.gpb.2018.08.002 (PMC6205082; doi:10.1016/j.gpb.2018.08.002)

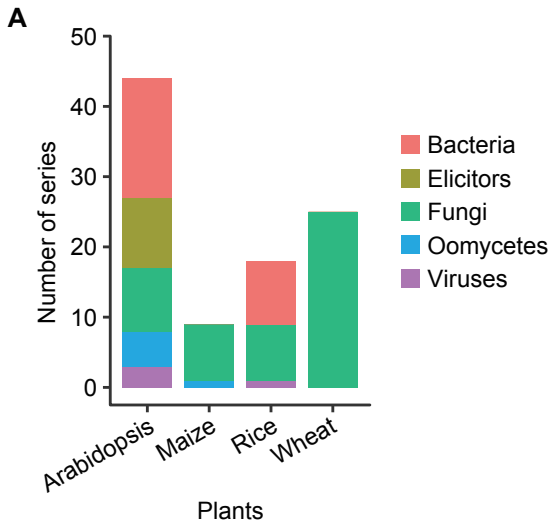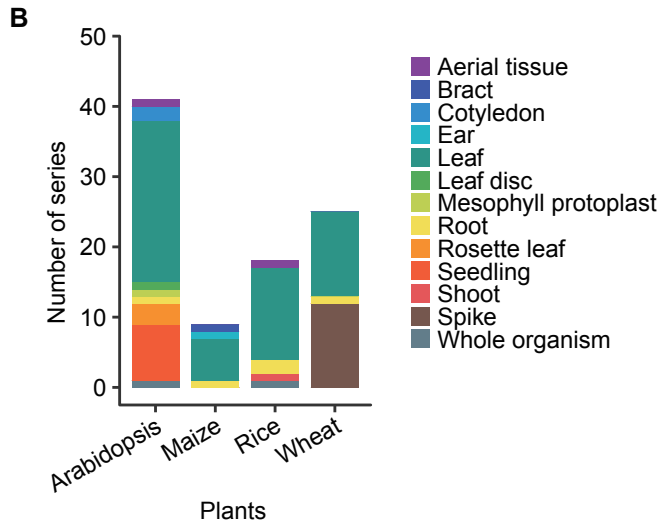

Supplement: Supplementary Figure S1 — Classifications of the microarray data A. The data were classified by pathogen types. B. The data were classified by plant tissues. [file mmc1.pdf]

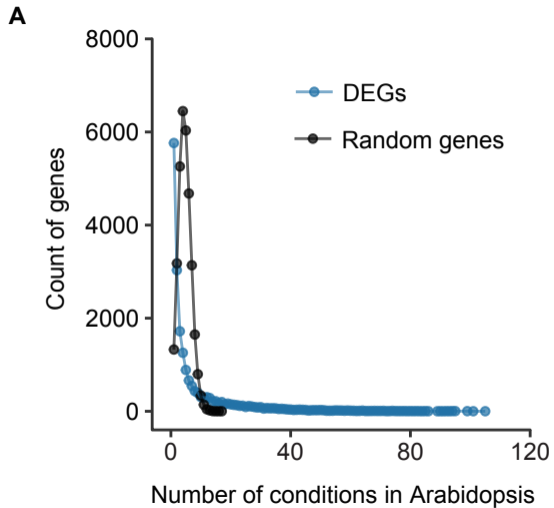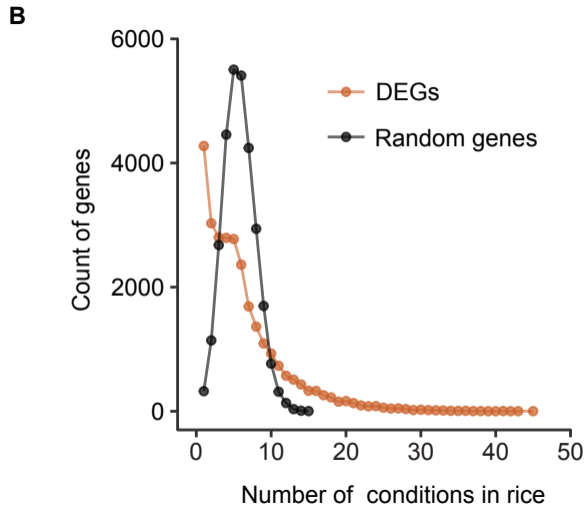

Supplement: Supplementary Figure S3 — The number of DEGs differentially expressed under multiple conditions is much larger than randomly expected A. Arabidopsis. B. Rice. [file mmc3.pdf]

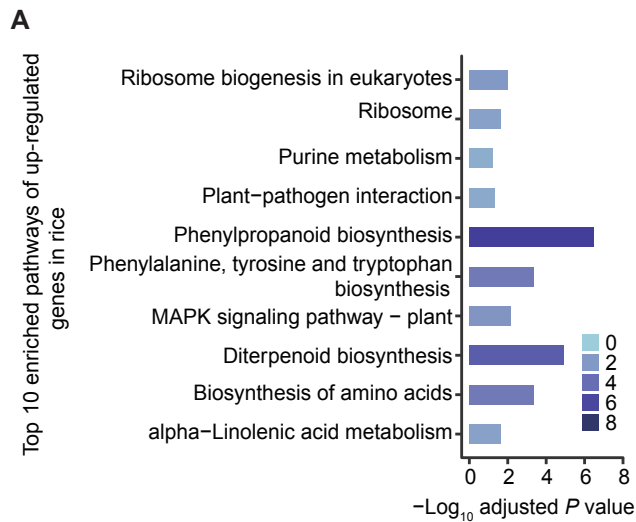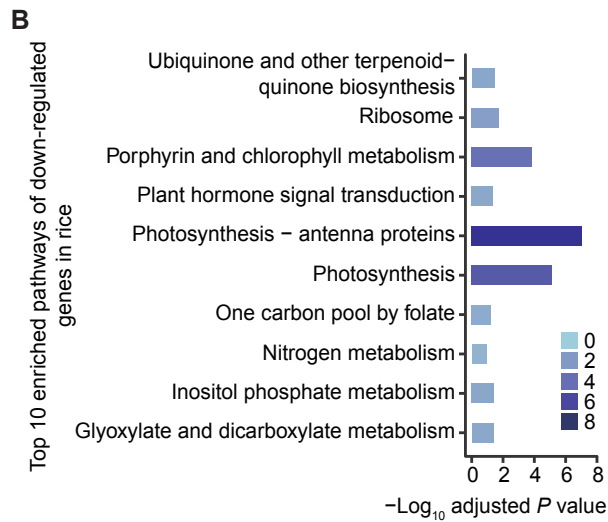

Supplement: Supplementary Figure S4 — Enriched KEGG pathways for rice A. Top 10 enriched KEGG pathways of 1496 consistently up-regulated freq_DEGs in rice under pathogen attacks (consistency_score ≥0.7). B. Top 10 enriched KEGG pathways of the 1144 consistently down-regulated freq_DEGs in rice under pathogen attacks (consistency_score ≤−0.7). [file mmc4.pdf]
