# Supplementary material for: PlaD: A Transcriptomics Database for Plant Defense Responses to Pathogens, Providing New Insights into Plant Immune System
Source: Genomics Proteomics Bioinformatics. 2018 Sep 26;16(4):283–93. doi: 10.1016/j.gpb.2018.08.002 (PMC6205082; doi:10.1016/j.gpb.2018.08.002)
Supplement: Supplementary Figure S2 — The co-expression sub-network of the gene AT1G56060 under the infection of Golovinomyces cichoracearum Genes co-expressed with AT1G56060 are shown in the left panel. The right panel shows enriched GO terms among the co-expressed genes. [file mmc2.pdf]

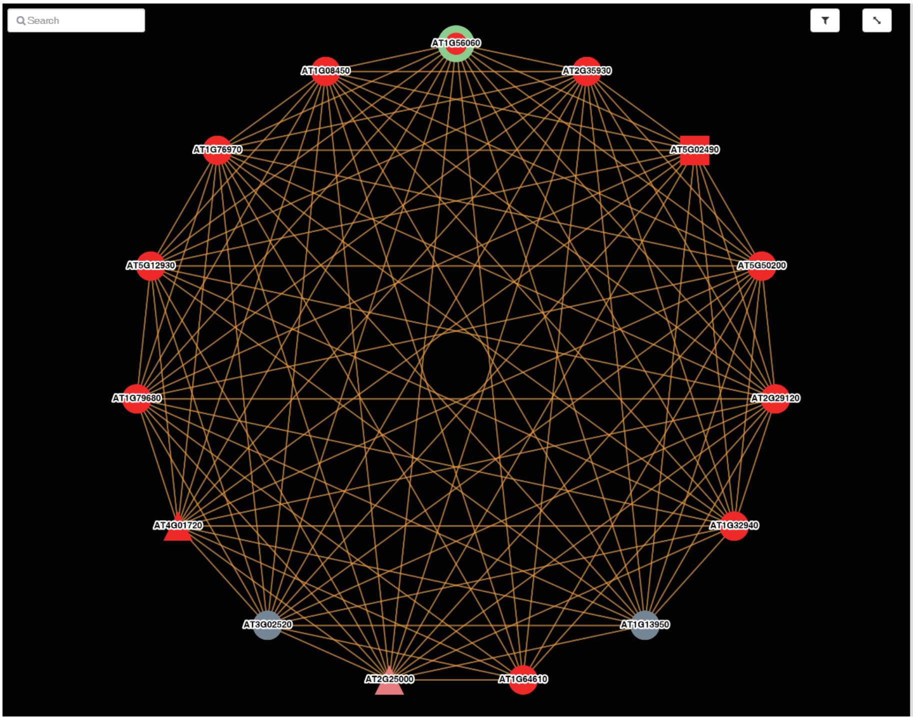

Gene type: Transcription factor Metabolic gene Others

DE type: Up Down Slightly_up Slightly_down Mixed Others

GO Enrichment Analysis of Co-expressed Genes with AT1G56060

GO-BP GO-MF GO-CC

Rank	GO Term	Adjusted *P* value
1	GO:0006182: cGMP biosynthetic process	0.00E+00
2	GO:0045905: positive regulation of translational termination	1.03E-05
3	GO:0045901: positive regulation of translational elongation	1.03E-05
4	GO:0006452: translational frameshifting	1.03E-05
5	GO:0055074: calcium ion homeostasis	1.93E-05
6	GO:0002679: respiratory burst involved in defense response	3.02E-05
7	GO:0046283: anthocyanin-containing compound metabolic process	5.67E-05
8	GO:0010204: defense response signaling pathway, resistance gene-independent	1.44E-04
9	GO:0009617: response to bacterium	1.58E-04
10	GO:0051865: protein autoubiquitination	1.63E-04
11	GO:0015706: nitrate transport	2.49E-04
12	GO:0002237: response to molecule of bacterial origin	2.95E-04
13	GO:0010167: response to nitrate	3.19E-04
14	GO:0009751: response to salicylic acid	3.78E-04
15	GO:0006874: cellular calcium ion homeostasis	3.93E-04
16	GO:0010089: xylem development	4.96E-04
17	GO:0006891: intra-Golgi vesicle-mediated transport	6.61E-04
18	GO:0009615: response to virus	8.38E-04
19	GO:0006457: protein folding	8.52E-04
20	GO:0042128: nitrate assimilation	8.99E-04
21	GO:0042742: defense response to bacterium	1.30E-03
22	GO:0031347: regulation of defense response	1.54E-03
23	GO:0009626: plant-type hypersensitive response	1.93E-03
24	GO:0009620: response to fungus	1.97E-03
